# Stem fixation techniques in revision total knee arthroplasty: A systematic review and meta‐analysis

**DOI:** 10.1002/jeo2.70086

**Published:** 2025-01-09

**Authors:** Francesco Onorato, Riccardo Giai Via, Francesco Bosco, Alessandro Dario Lavia, Luca Barberis, Marcello Capella, Alessandro Massè, Salvatore Risitano

**Affiliations:** ^1^ Department of Orthopaedics, Traumatology and Rehabilitation University of Turin Turin Italy; ^2^ Department of Precision Medicine in Medical, Surgical and Critical Care (Me.Pre.C.C.) University of Palermo Palermo Italy; ^3^ Department of Orthopaedics and Traumatology G.F. Ingrassia Hospital Unit Palermo Italy; ^4^ Department of Economics Boston College Boston Massachusetts USA; ^5^ Department of Orthopaedic Surgery and Traumatology Città della Salute e della Scienza Turin Italy

**Keywords:** cement, cementless, meta‐analysis, revision TKA, stem fixation, total knee arthroplasty

## Abstract

**Purpose:**

This systematic review and meta‐analysis aimed to compare the clinical and radiological outcomes of patients undergoing revision total knee arthroplasty (rTKA) using uncemented press‐fit stems (hybrid fixation) versus cemented stems (cemented fixation). It is also examined whether cemented fixation offers any superiority over hybrid fixation regarding implant survival, clinical function, imaging analysis and complication rates.

**Methods:**

Following the PRISMA guidelines, a systematic review and meta‐analysis were conducted on five databases (Pubmed, Scopus, Embase, Medline and Cochrane). Articles were evaluated according to levels of evidence (LoE). Retrospective studies were analysed with risk of bias in nonrandomised studies of interventions (Robins‐I) and randomised controlled trials with risk of bias 2 (RoB‐2). This review was registered in the International Prospective Register of Systematic Reviews database. Meta‐analysis was performed using R software, with *p* < 0.05 considered statistically significant.

**Results:**

Data from 12 comparative studies with 1303 patients (1352 rTKAs) were analysed. Survival rates of hybrid and cemented fixations were comparable, with a significant trend favouring hybrid fixation (*p* = 0.04). Infection and aseptic loosening were the most common causes of failure. Radiographic failure rates showed no significant differences between fixation methods (*p* = 0.4). Meta‐analysis indicated better results with hybrid fixation, although not statistically significant (KSS functional *p* = 0.15; KSS clinical *p* = 0.5). High heterogeneity was observed due to variations in patient characteristics and surgical strategies.

**Conclusion:**

Both hybrid and cemented fixation techniques achieve satisfactory clinical results in rTKA, with hybrid fixation demonstrating an overall lower failure rate. The choice of fixation method must be tailored to individual patient characteristics and surgical considerations. Further high‐quality randomised trials are needed to refine these results and optimise fixation strategies to improve patient outcomes.

**Level of Evidence:**

Level IV.

**Abbreviations**
BMI: body mass index
CI: confidence interval
KOOS: Knee Injury and Osteoarthritis Outcome Score
KSS: Knee Society Score
LoE: levels of evidence
OR: odds ratio
PRISMA: Preferred Reporting Items for Systematic Reviews and Meta‐Analyses
RCT: randomised controlled trial
RLLs: radiolucent lines
RoB‐2: risk of bias 2
ROBINS‐I: risk of bias in nonrandomised studies of interventions
rTKA: revision total knee arthroplasty
SMD: standardised mean differences
TKA: total knee arthroplasties

## INTRODUCTION

With an aging population and a growing demand for an active lifestyle, the number of primary total knee arthroplasties (TKAs) has significantly increased [2, 19]. Despite advancements in design and materials extending the longevity of implants, approximately 3%–5% of all TKAs fail annually due to septic or aseptic loosening or component failure [1, 19]. Given the rise in primary replacements and the decreasing median age for prosthetic implantation, the incidence of revision TKAs (rTKAs) is projected to increase by 88% by 2050 [2, 19, 33]. However, revision TKA is more expensive, technically challenging and less satisfying than primary TKA; satisfaction rate is reported to differ from 65% to 85%, respectively [1]. Therefore, optimal surgical management, implant selection and fixation methods are crucial for improving function and cost‐effectiveness [2, 19, 33].

National registry data from 11 countries across four continents show that aseptic loosening, infection, instability, patellofemoral complications and pain are major causes of rTKA with incidence varying from 27.9% for aseptic loosening to 5.1% for patellofemoral complications [16]. Although successful revision can improve joint function, problems arise due to deformity, poor bone quality and bone defects, making revision implants more challenging [16].

There are different surgical philosophies for approaching rTKA, especially introducing new versatile biomaterials [2, 13, 16, 19]. Wedge or block augmentations and bone grafts are used with cement to compensate for epiphyseal fixation, while metaphyseal sleeves or trabecular metal cones are used to improve metaphyseal fixation. These devices enable load transfer to avoid stress shielding, allowing the use of shorter diaphyseal stems for secure fixation [13, 32].

During rTKA procedures, epiphyseal and metaphyseal areas may be inadequate for proper implant fixation. Consequently, it is widely accepted that diaphyseal intramedullary stems secure and improve implant fixation by offloading the metaphysis, where augmentation may be needed.

Historically, two methods of diaphyseal fixation have been used: cemented or press‐fit stems. Both implants present specific strengths and weaknesses [15, 17, 29, 32]. Uncemented press‐fit stems are generally longer, offloading the metaphysis with optimal rotational stability and better alignment of the intramedullary axis [15, 32]. However, pain related to high contact pressures between the stem tip and the diaphyseal bone has been described [17, 29]. On the other hand, fully cemented stems can reduce stem length and contact stresses. In addition, antibiotic‐loaded cement can be used. However, the use of cement prolongs operative time and complicates component removal in case of re‐revision, especially during the removal of cement fragments from intramedullary canal [30].

The literature is still controversial, as no study has yet demonstrated the superiority of one fixation system over the other in terms of clinical outcomes, radiographic evaluation or implant survival. Previous meta‐analyses have suggested that both fixation systems have similar reintervention rates and failures in radiographic evaluation [28, 40], although a tendency toward a less invasive juxta‐articular fixation through a stable metaphyseal fixation and cemented short stems seemed to emerge from clinical studies and recent systematic reviews [36].

The aim of this systematic review and meta‐analysis is to examine the clinical and radiological outcomes of patients undergoing rTKA with uncemented press‐fit stems (hybrid fixation) and compare them with the outcomes of patients undergoing rTKA with cemented stems (cemented fixation). Furthermore, we hypothesise that cemented fixation is not superior to hybrid fixation regarding implant survival, clinical function, imaging analysis and complications.

## MATERIALS AND METHODS

### Research question

This study adhered to the Preferred Reporting Items for Systematic Reviews and Meta‐Analyses (PRISMA) guidelines [25]. Two authors (R. G. V. and F. O.) independently searched and evaluated the articles to avoid bias and, furthermore, participate to manuscript writing. A third author (F. B.) was consulted to resolve any doubts regarding contents and methodology. The Patient, Intervention, Comparison, Outcomes and Study design was used to classify and answer clinical questions according to the PRISMA checklist: patient (P); intervention (I) patients who underwent revision total knee replacement with hybrid method; comparison (C), patients who underwent revision total knee replacement with cemented method; outcomes (O) clinical, radiographic outcomes and complication rates; study design model (S) retrospective and randomised controlled trial (RCT) studies.

### Inclusion and exclusion criteria

Inclusion criteria for the reviewed studies were articles about patients undergoing revision total knee replacement with either a cemented or hybrid method of at least one stem component, written in English, about human subjects, published between 2003 and November 2023 with a minimum mean follow‐up of at least 36 months, RCTs, prospective and retrospective case‐control studies with Level of Evidence (LoE) 1–4 [5]. Biochemical and in vitro studies, case reports, editorials, book chapters, technical reports, preclinical studies and review articles were excluded from the search. Studies with LoE five or lower were also excluded for better‐quality research.

### Search strategy and study screening

**Figure 1:**
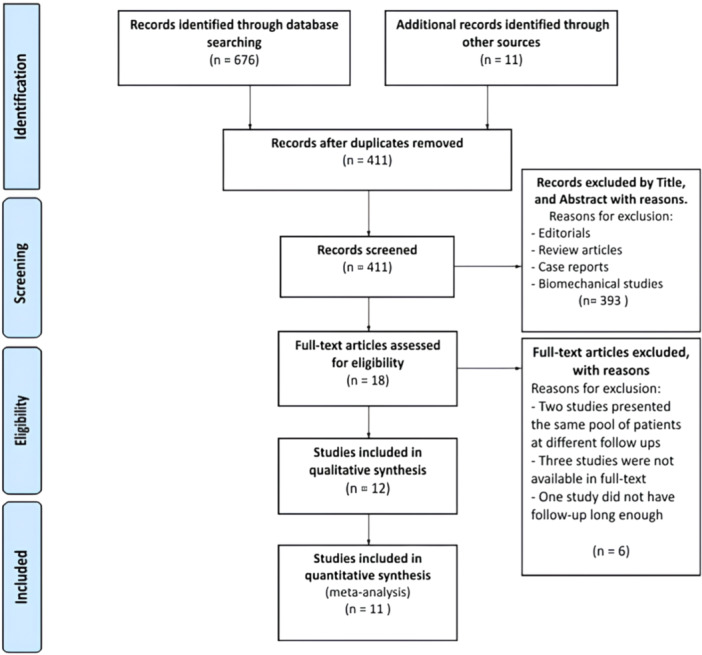
Preferred Reporting Items for Systematic Reviews and Meta‐Analyses (PRISMA) flowchart.

We performed a literature search in five databases (Pubmed, Scopus, Embase, Medline and Cochrane) with the following MeSH terms: ((Revision) AND (Knee Prosth* Revision) AND (Knee Arthroplast* Revision)) AND ((stem) AND (cemented) OR (hybrid) OR (biological fixation)). The search included studies published from 2003 to November 2023. With the above MeSH terms, we found a total of 687 studies. After the exclusion of duplicates, 411 studies were included. After reviewing the titles and abstracts of the studies, 393 were excluded, leaving 18 eligible studies. Following a full‐text evaluation for eligibility based on the exclusion and inclusion criteria, 12 studies were selected for qualitative analysis, while 11 were evaluated through quantitative analysis. The included studies directly compared the functional and radiographic outcomes and the differences in complication rates between patients undergoing revision TKA using either a hybrid or cemented approach. Additionally, the bibliography of each article was reviewed to identify further relevant studies. The PRISMA flowchart detailing the study selection process is presented in Figure 1.

### Methodological quality assessment

**Figure 2:**
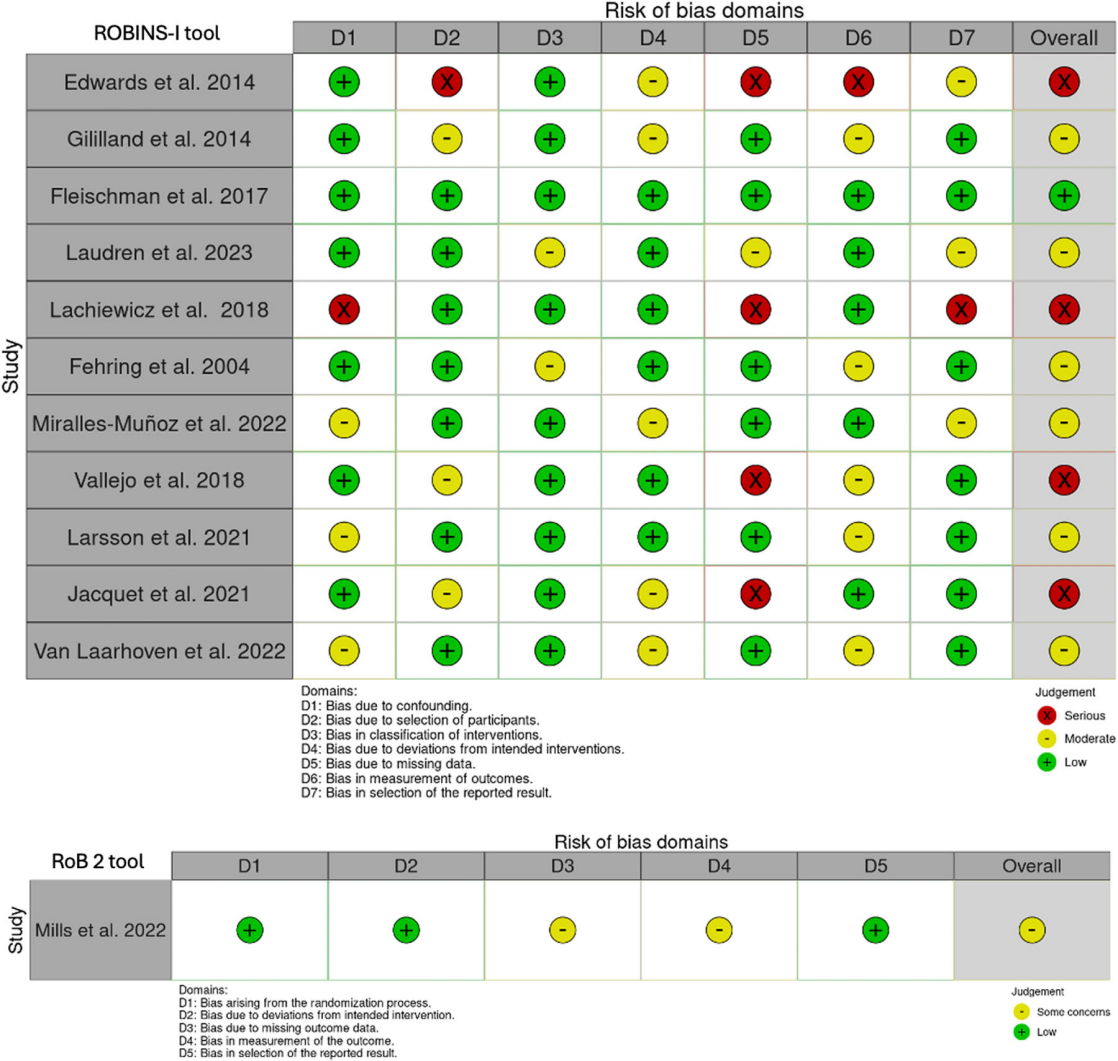
Methodological quality of the included studies according to the risk of bias in nonrandomised studies of interventions (ROBINS‐I) tool and risk of bias 2 (RoB‐2) tool.

Each included article underwent classification based on the Oxford Centre for Evidence‐Based Medicine 2011. With this tool, articles were classified from 1 to 5, where LoE 1 represented a better design, methodological quality and lower risk of bias in the study under review. The Robins‐I tool for assessing the risk of bias in nonrandomised studies of interventions was employed for retrospective studies [10, 11]. The methodological quality of the RCT article was evaluated by the Risk of Bias 2 (Figure 2) [38]. Two authors (R. G. V. and F. O.) used these tools, with one author (F. B.) consulted to resolve uncertainties. This systematic review and meta‐analysis were registered on the International Prospective Register of Systematic Reviews in November 2023 [37].

### Data extraction

The data extracted from the included articles were systematically recorded on a template, which comprised the following elements: author and publication year, study design, patient sample size, mean age of the participants, mean Body Mass Index, survival rates, cause of failures, imaging failures, rates of complications and revisions, details regarding the type of implant used for revision total knee replacement, pre and postsurgery subjective scores like Knee Society Score (KSS), Knee Injury and Osteoarthritis Outcome Score (KOOS) or Western Ontario and McMaster Universities Osteoarthritis Index (WOMAC) and Range of Motion (ROM). This organised data extraction and analysis approach facilitated a comprehensive understanding of the study findings.

### Data analysis

A comprehensive meta‐analysis of preoperative and postoperative outcomes of cemented versus hybrid rTKAs has been conducted. KSS score has been considered in this analysis, comparing the score's clinical and functional characteristics. The analysis combined the data as standardised mean differences (SMD), using random‐effect analysis and inverse weighting for pooling. The average effect size and a 95% confidence interval have been computed via the Jackson method. The Cochran's Q test and Higgins' *I*² statistics have been performed to check for heterogeneity between studies. The SMD required a *p* value of 0.05 to be considered statistically significant. Funnel plots and Egger's tests have been performed to test for eventual publication bias. The Mantel–Haenszel Method was applied to calculate the odds ratio (OR) to compare the radiographic failure, overall survival and the failures due to aseptic loosening and infection to obtain a weighted estimate under a fixed‐effects model. The statistical analysis was performed by a professional statistician (A. L.) using R software, version 4.1.3 (2022, R Core Team).

## RESULTS

This comprehensive analysis included data from 12 comparative studies [7, 9, 10, 12, 13, 17, 20, 21, 22, 23, 30, 31] encompassing 1303 patients who underwent 1352 rTKAs. Within this population, 1377 stems were implanted with a hybrid fixation technique, while 959 were implanted using cemented fixation. All included studies focused on the medium‐to‐long‐term follow‐up results of fixation, with a minimum follow‐up period of 3.7 years. Ten studies reported follow‐up results over 5 years (range 3.7–10 years) [7, 12, 13, 17, 20, 21, 22, 23, 30, 31].

Regarding the study design, 10 studies were retrospective case–control studies [7, 9, 10, 12, 13, 17, 20, 21, 22, 23], one was a patient‐blinded RCT [30] and one was a prospective nonrandomised comparative study [31]. Except for Fehring et al. [9], all studies were published within the last 10 years. Table 1 presents a concise summary of the main demographic characteristics of the included studies.

**Table 1:** Details of the included studies in the systematic review.

Study and publication year	Study design	Patients		Number of stems		Mean follow‐up (months)		Mean age		Male %		Female %		BMI		Constraint %		Lost to follow‐up
		C	H	C	H	C	H	C	H	C	H	C	H	C	H	C CCK; RHK CPS; PS	H CCK; RHK CPS; PS	(%)
Edwards et al. 2014 [7]	Retrospective case‐control	51	63	102	126	45	52	65	65	51	51	49	49	/	/	/	/	24
Gililland et al. 2014 [12]	Retrospective case‐control	49	32	98	64	76	121	65	64	49	34	51	66	/	/	24; 2	94; 0	/
																0; 71	0; 6	
Mills et al. 2022 [30]	RCT	10	10	20	20	120	120	74.9	72.2	50	10	50	90	/	/	100; 0	100; 0	10
																0; 0	0; 0	
Fleischman et al. 2017 [10]	Retrospective case‐control	54	158	108	316	64.3	59.6	65.8	63.9	30.6	42.7	69.4	57.3	32.1	32.8	83;/	91.5;/	/
																/;/	/;/	
Laudren et al. 2023 [23]	Retrospective case‐control	23	42	45	85	88.8	85.2	67.3	69.2	43	53	47	57	29.2	29.2	0; 0	92; 0	10
																96; 4	0; 4	
Lachiewicz et al. 2018 [21]	Retrospective case‐control	31	47	34	50	72	72	68	68	42	40	58	60	33	33	47; 0	52; 0	21
																0; 53	0; 48	
Fehring et al. 2004 [9]	Retrospective case‐control	109^a^		107	95	53	61	67	68	/	/	/	/	/	/	46; 0	34; 0	/
																0; 54	0; 66	
Miralles‐Muñoz et al. 2022 [31]	Prospective nonrandomised	31	42	31	42	76	76	67.8	65.3	38.8	40.5	61.2	59.5	30.1	31.8	0; 0	0; 0	5
																0; 100	0; 100	
Vallejo et al. 2018 [13]	Retrospective case‐control	29	38	58	76	84	84	79.7	78.3	/	/	/	/	31.1	31.2	/	/	21
Larson et al. 2021 [22]	Retrospective case‐control	63	47	63	47	60	60	65.9	66	52.4	42.6	47.6	57.4	/	/	/	/	/
Jacquet et al. 2021 [17]	Retrospective case‐control	33	66	66	132	104	110	72.9	72.6	/	/	/	/	28.1	28	0; 100	0;100	20
																0; 0	0; 0	
Van Laarhoven et al. 2022 [20]	Retrospective case‐control	100	148	227	323	50.4	92.4	66.5	64.6	31	33.8	69	66.2	29.1	29.3	0; 100	0;100	/
																0; 0	0; 0	

*Note*: /, not clarified; %, number reported are percentage of the total.

Abbreviations: BMI, body mass index; C, cement fixation; CCK, constrained condylar knee; CPS, constrained posterior stabilised H, hybrid fixation; PS, posterior stabilised; RCT, randomised controlled trial; RHK, rotating hinge knee.

^a^ Specific group subdivision was not given.

The reasons for revision differed among the studies. Therefore, the etiologies for rTKA were categorised as ‘septic’ or ‘aseptic’, as detailed in Table 2. Two studies analysed only the diaphyseal fixation of the tibial component [22, 31], while Lachiewicz et al. focused solely on the femoral component fixation [21].

**Table 2:** Study reasons of revision defined as ‘septic’ or ‘aseptic’.

Study and publication year	Only aseptic	Only septic	Septic and aseptic
Edwards et al. 2014 [7]		X	
Gililland et al. 2014 [12]	X		
Mills et al. 2022 [30]	X		
Fleischman et al. 2017 [10]			X
Laudren et al. 2023 [23]			X
Lachiewicz et al. 2018 [21]			X
Fehring et al. 2004 [9]	X		
Miralles‐Muñoz et al. 2022 [31]	X		
Vallejo et al. 2018 [13]	X		
Larson et al. 2021 [22]			X
Jacquet et al. 2021 [17]	X		
Van Laarhoven et al. 2022 [20]			X

Seven studies [10, 17, 20, 21, 23, 30, 31] evaluated and reported bone defects during rTKA according to the Anderson Orthopaedic Research Institute classification. Of these, only four papers [12, 20, 21, 23] also described the type of metaphyseal fixation used for metaphyseal defects.

**Figure 3:**
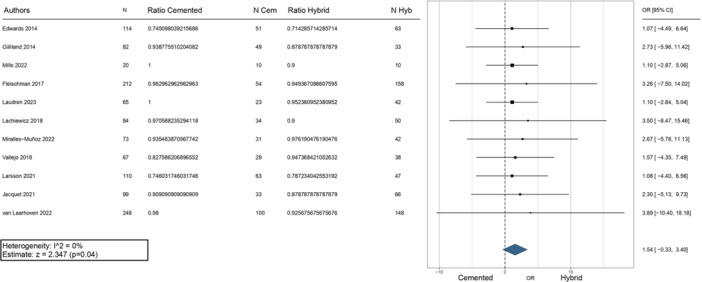
Meta‐analysis evaluating the survival rate between cemented and hybrid rTKA. Cem, cemented; CI, confidence interval; Hyb, hybrid; N, number of evaluation cases; OR, odds ratio; rTKA, revision total knee arthroplasty.

The survival rate of the two fixation methods was comparable, ranging from 71.6% at 4 years to 98% at more than 6 years for the hybrid approach and from 75% at 4 years and 100% at 10 years with cemented fixation (Table 3). The meta‐analysis indicated a statistically significant trend favouring the hybrid approach (*p* = 0.04) (Figure 3). Only Van Laarhoven et al. [20] reported a significant difference in favour of the fully cemented fixation at 10‐year follow‐up. In contrast, all the other authors did not find significant differences in implant survival at medium‐term follow‐up, with only three studies reporting a higher survival rate in the case of hybrid fixation [13, 22, 31].

**Table 3:** Studies with major complications or radiographic failure following revision with different stem fixation techniques.

Study and publication year	Aseptic loosening		Infection		Instability		Fracture		Imaging evidence of failure		Implant survival rate		
	C	H	C	H	C	H	C	H	C	H	C (%)	H (%)	*p* Value
Edwards et al. 2014 [7]	3	3	10	15					5	2	75	71.4	>0.05
Gililland et al. 2014 [12]			1	2	2	1	0	1	3	3	93	91	>0.05
Mills et al. 2022 [30]	0	0	0	1	2	1			0	0	100	90	>0.05
Fleischman et al. 2017 [10]	7	14	8	18					2	8	96.5	95	>0.05
Laudren et al. 2023 [23]	0	4	9^a^						0	10	100	95.6	>0.05
Lachiewicz et al. 2018 [21]	1	5							3	8	96.7	90	>0.05
Fehring et al. 2004 [9]	0	4							0	10	100	96	/
Miralles‐Muñoz et al. 2022 [31]	1	1	1	0					8	8	94	98	>0.05
Vallejo et al. 2018 [13]			3^a^		2^a^		1^a^		6	5	84	94	=0.61
Larson et al. 2021 [22]											74.6	78.7	>0.05
Jacquet et al. 2021 [17]			10^a^		2^a^						90.9	87.75	/
Van Laarhoven et al. 2022 [20]	19^a^		23^a^				4^a^				98.5	92.7	<**0.05**

Abbreviations: C, cement fixation; H, hybrid fixation.

^a^ Specific group subdivision was not given.

**Figure 4:**
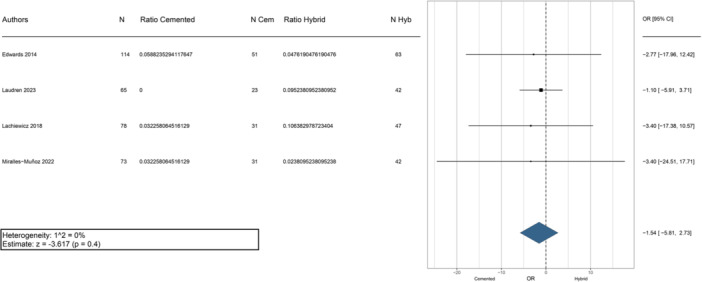
Meta‐analysis evaluating failures due to infection between cemented and hybrid rTKA. Cem, cemented; CI, confidence interval; Hyb, hybrid; N, number of evaluation cases; OR, odds ratio; rTKA, revision total knee arthroplasty.

**Figure 5:**
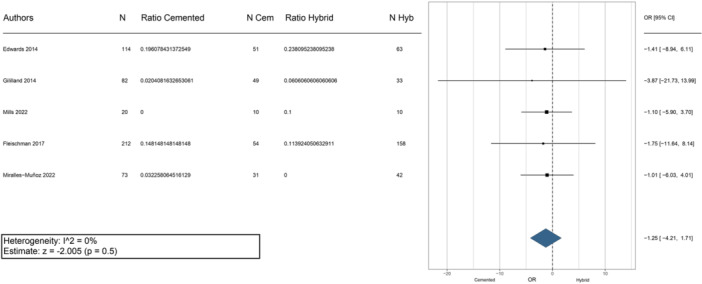
Meta‐analysis evaluating failures due to aseptic loosening between cemented and hybrid rTKA. Cem, cemented; CI, confidence interval; Hyb, hybrid; N, number of evaluation cases; OR, odds ratio; rTKA, revision total knee arthroplasty.

Infection and aseptic loosening were the most common causes of failure requiring reoperation. The meta‐analysis of the five studies evaluating failures due to infection showed no significant difference between the fixation methods (Figure 4). Similarly, the meta‐analysis of failures due to aseptic loosening found no significant differences between the two fixation methods, as shown in the forest plot in Figure 5.

**Figure 6:**
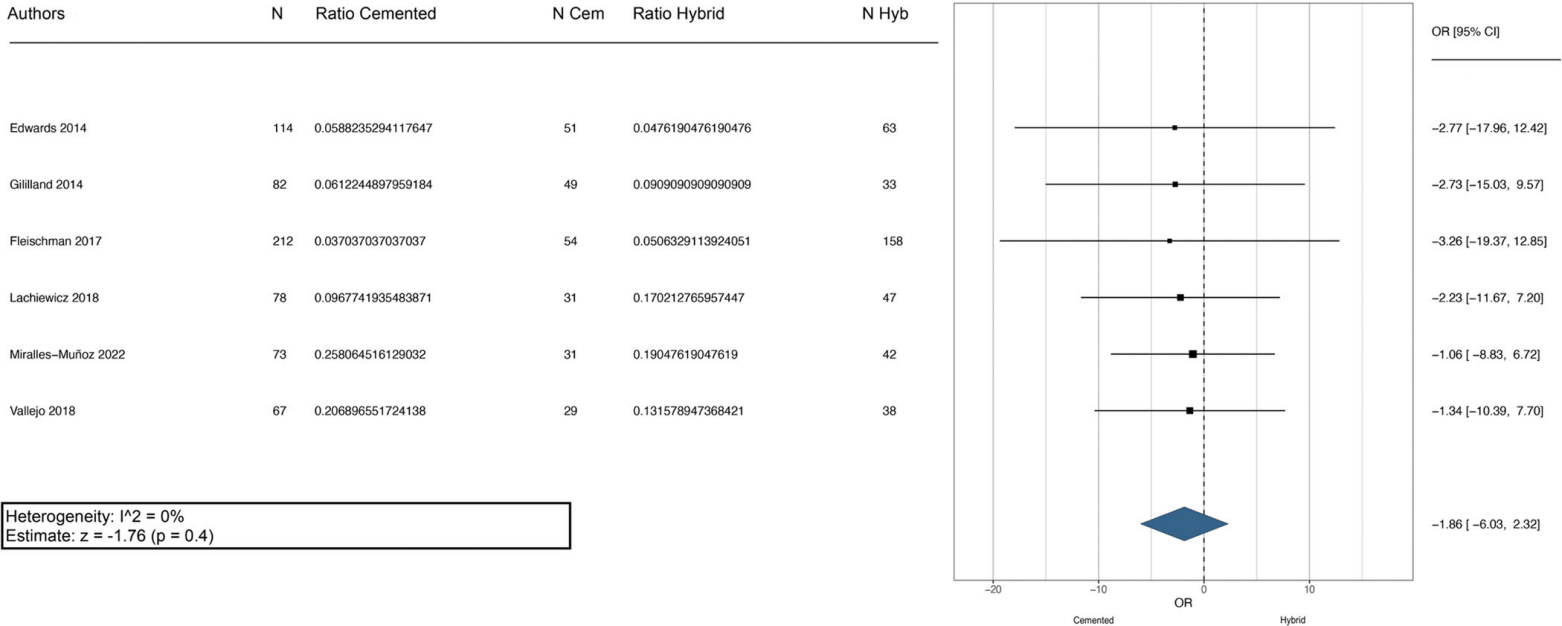
Meta‐analysis evaluating radiographic failure between cemented and hybrid rTKA. Cem, cemented; CI, confidence interval; Hyb, hybrid; N, number of evaluation cases; OR, odds ratio; rTKA, revision total knee arthroplasty.

Nine studies reported radiographic evidence of failure [7, 9, 10, 12, 13, 21, 23, 30, 31]. Within the cemented fixation group, 27 out of 683 stems were considered radiographically loose, while in the hybrid fixation group, 54 out of 1060 press‐fit stems showed radiographic failure. Table 3 provides a detailed summary of the major complications and radiographic failures for each study. The weighted OR comparing radiographic failure between cemented and hybrid rTKA showed a nonsignificant tendency in favour of the cemented approach (Figure 6).

**Figure 7:**
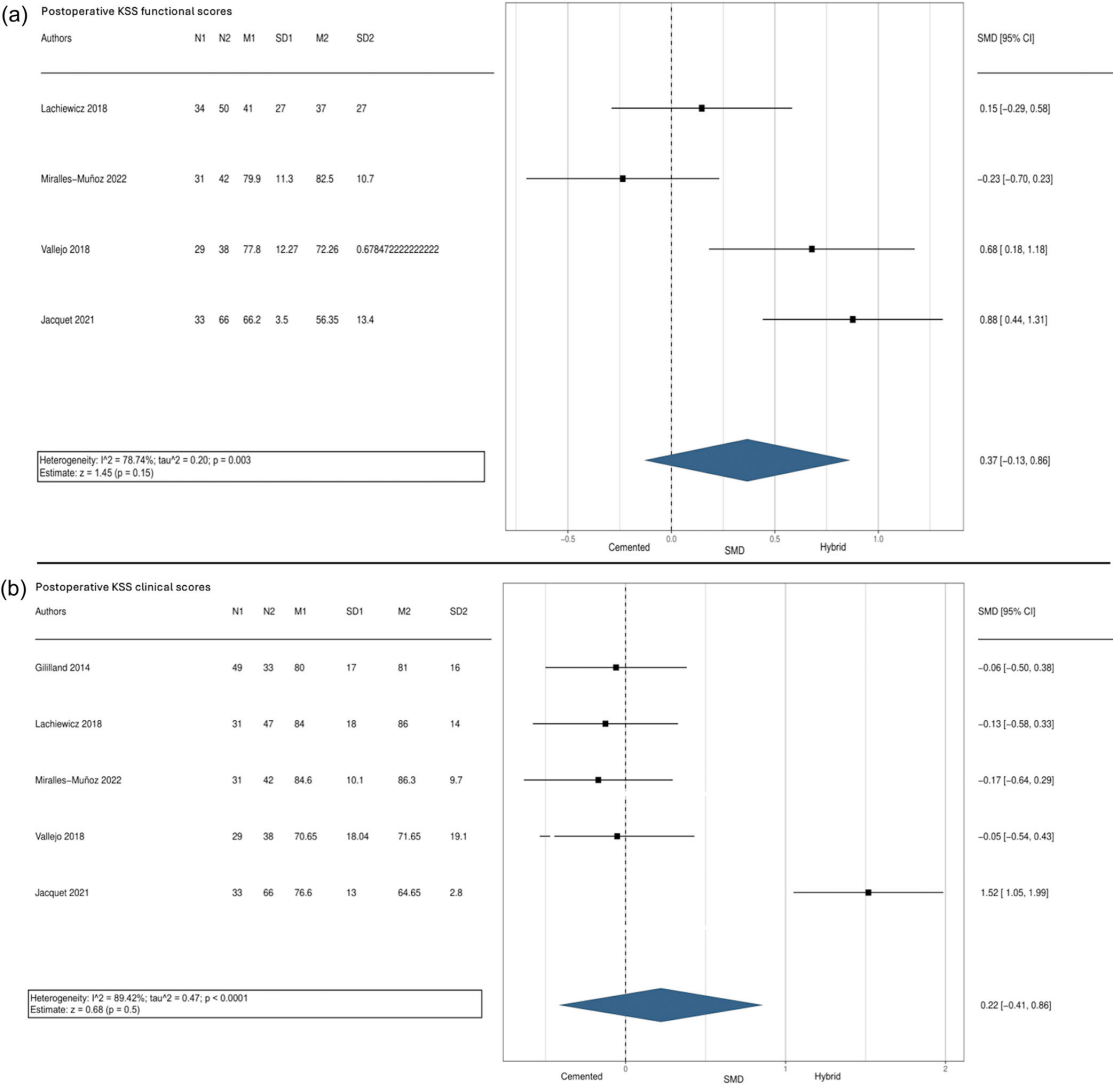
Meta‐analysis of KSS functional (a) and clinical (b) scores between cemented and hybrid rTKA. Cem, cemented; CI, confidence interval; Hyb, hybrid; KSS, Knee Society Score; N, number of evaluation cases; OR, odds ratio; rTKA, revision total knee arthroplasty.

Despite the high heterogeneity among the studies, the meta‐analysis conducted for KSS functional and clinical scores indicated better outcomes after rTKA with the hybrid approach than the cemented approach. However, these results were not statistically significant (KSS functional *p* = 0.15; KSS clinical *p* = 0.5), as demonstrated in Figure 7. Furthermore, the funnel plots indicated a moderate publication bias concerning both overall failure and KSS functional and clinical score.

## DISCUSSION

The main finding of the present systematic review and meta‐analysis is that both fixation techniques achieve satisfactory clinical results in rTKA with evidence of an overall lower failure rate of hybrid fixation when compared to cemented. High heterogeneity was observed among studies due to variations in patients' characteristics and surgical strategies, which prevented drawing an evidence‐based conclusion on the best fixation technique. Furthermore, as detailed in Table 2, the surgical indications for rTKA were not homogeneous, as 5/11 studies did not distinguish between TKA failures due to aseptic and septic causes. These differences could impact the overall survival analysis because, as Fleischman et al. showed, the risk of mechanical failure after initial revision for periprosthetic joint infections is higher [10]. This finding is in accordance with Edwards et al. [7], reporting the highest re‐revision for recurrent sepsis (25/114) and the lowest survival rates among the studies included, possibly due to the inclusion of only infection cases.

The overall survival rate showed an evidence of the superiority in favour of the hybrid approach. However, Mills et al., within their RCT, reported the only one failure in the hybrid group at 10‐year follow‐up despite the small study cohort. Similarly, in their original study, Heesterbeek et al. reported more complications within the hybrid group at a 2‐year follow‐up [15, 30].

Despite the complexity and the compromise surgeons sometimes face, rTKA could be considered a successful treatment in adequately selected cases when performed by experienced hands [27, 34].

As a secondary outcome, the KSS postoperative score from this meta‐analysis suggested that the hybrid fixation technique could achieve satisfactory and even better clinical outcomes than cemented fixation. A possible explanation may reside in the ease through which press‐fit stems follow the intramedullary axis of bony canals, therefore, perfectly respecting patient native axis.

A previous meta‐analysis performed by Wang et al., which included only studies up to 2014 [40], concluded that an ideal stem fixation system in revision TKA could not be determined with the previously available evidence. The authors reported a similar complication rate for both fixation methods, but with an extended follow‐up period. They noted an increased loosening with cemented fixation rather than cementless [40]. A meta‐analysis carried out by Sheridan et al. excluding on purpose the available literature before 2010 provide an estimate of the modern stem performances [36], concluding that hybrid stem fixation afforded a lower overall failure rate when compared to cemented stems. Starting from this evidence, we decided to include all the available literature of the last 20 years, but to confine the analysis only to comparative studies.

It is well‐accepted within the orthopaedic community that rTKA is expensive and challenging due to bone defects that make fixation troublesome even for experienced surgeons [18]. Despite recent advances in meta‐epiphyseal fixation, intramedullary stems play a fundamental role in the stability and correct alignment of the revised implant [18, 39]. Furthermore, intramedullary stems at least 70 mm long have been shown to relieve the deficient epiphyseal bone of approximately 30% of the axial load, with positive effects, especially in patients with marked juxta‐articular osteoporosis [9].

Historically, studies reported optimal stability and increased flexibility of prosthetic component placement with cemented stems [9, 27]. Cemented fixation was advocated due to reduced micro‐motion and migration of implants over time and the possibility of antibiotic loading. Additionally, cemented stems can be undersized, making them easier to centre in the medullary canal and better adapted to metaphyseal fixation without stem offsets [35]. In contrast to these theories, our study showed that most orthopaedic surgeons still preferred to perform revision TKA with cementless stems, as depicted in Table 1.

The author's hypothetical reasons and motivations for this preference lie in easier component removal during an eventual re‐revision and the certainty of implanting the stem along the correct anatomical axis of the bone without the risk of subsequent epiphyseal component malpositioning. Some authors have also suggested that hybrid fixation reduces stress shielding in the bone surrounding the stem, thus lowering the risk of aseptic loosening [1, 6, 39]. This systematic review compared the incidence of radiographic failures between hybrid and cemented fixation, considering it a factor that could predict clinical failure, even though few studies reported a positive correlation between radiolucent lines (RLLs) and subsequent clinical loosening [41]. Indeed, several studies demonstrated that early development of RLLs secondary to surgical technique, either due to inadequate cement penetration in sclerotic bone or component malpositioning, did not later evolve into clinical symptoms. However, all the studies included in this analysis have at least 4 years of follow‐up. Therefore, the presence of RLLs was considered a premonitory sign of aseptic loosening. The meta‐analysis regarding imaging failure rate revealed no significant differences between the two fixation methods, although a higher frequency of RLLs was noted in the hybrid fixation group. However, it is worth mentioning that studies reporting imaging outcomes utilised different evaluation systems. Most authors [7, 10, 12, 13, 23] assessed radiographic loosening according to the modified KSS proposed by Fehring et al. [9] or the updated version proposed by Meneghini et al. [26, 28]. Two studies [21, 31] utilised the radiographic Knee Society scoring system proposed by Ewald et al. [8, 28].

In contrast, one study used a specific radiosterometric analysis for micromotion [30] and correlated this progression with clinical loosening. Infection and aseptic loosening were the most common complications based on our analysis, without significant differences between the two groups. In contrast to previous reports in the literature [4], our analysis showed that cemented fixation did not outperform hybrid fixation concerning aseptic loosening. A recent study showed that septic rTKA had a higher infection rate than aseptic rTKA even when two‐stage revision was performed [24]. Therefore, septic complications could be a consequence of an unhealed infectious state. Six studies [7, 9, 10, 12, 17, 20] reported using antibiotic‐loaded cement for the cemented fixation group. However, the effectiveness of low‐dose prophylactic antibiotic‐loaded cement has been controversial due to inconclusive results and the potential for bacterial resistance [3]. Jacquet et al. probably interpreted most surgeons' opinions, finding that a short‐cemented tibial stem offers an identical survival rate to a long uncemented stem, but only when combined with trabecular metal metaphyseal cones [17]. Another consideration concerning this topic relates to surgeon choices based on specific patient bone quality, which might induce using a cemented stem in cases of serious osteopenia while reserving hybrid fixation for cases with well‐preserved bone cortices.

Further developments in stem osteo‐inductive and osteo‐conductive properties might decrease stress shielding and improve cementless fixation techniques, positively affecting bearing surfaces [14].

This meta‐analysis presents some limitations which prevent robust evidence of the results. First, there is only one study with a high LoE, while most others are retrospective case–control studies. Second, as already mentioned and confirmed by the funnel plots, there was high heterogeneity among the studies due to variations in patient demographics, surgical indications, different bone defects and meta‐epiphyseal fixation strategies. Additionally, many studies reported various levels of constrained bearings and polyethylene insert designs, which affect the stress transferred to the stems, especially in hybrid fixation. It is well‐accepted that at least two bone fixation areas should be addressed for adequate implant stability [7, 27]. A more detailed description of meta‐epiphyseal fixation and constrained bearings would have allowed for a clearer understanding of the specific contribution of the stems alone. Furthermore, three studies compared patients who underwent revision TKA of a single component due to aspetic loosening [1, 20, 21, 29]. Nevertheless, this comprehensive meta‐analysis stands out due to its unique comparative design, the substantial number of index knees and the contemporary analysis of overall implant survival, aseptic loosening, infection rate, radiographic failure and clinical outcomes based on the latest available clinical evidence.

## CONCLUSION

Based on the available literature, no definitive evidence in revision TKA favoured one technique over the other regarding clinical function or complications. Hybrid fixation demonstrated an overall lower failure rate, although high heterogeneity was observed among studies. The results suggest that hybrid and cemented fixation should be tailored to individual patient characteristics and surgical considerations, with both techniques remaining viable options for rTKA. Further high‐quality randomised trials are needed to refine these findings and optimise fixation strategies to improve patient outcomes.

## AUTHOR CONTRIBUTIONS

Francesco Onorato and Riccardo Giai Via have contributed substantially to conception and design, data acquisition, analysis and interpretation. They agree to be accountable for all aspects of the work in ensuring that questions related to the accuracy or integrity of any part of the work are appropriately investigated and resolved. Alessandro Dario Lavia, Luca Barberis and Marcello Capella have contributed substantially to the data analysis, interpretation and manuscript drafting. Francesco Bosco and Alessandro Massè have significantly contributed to revising the manuscript critically for important intellectual content, giving final approval of the version to be published. Salvatore Risitano has made substantial contributions to concept and design.

## CONFLICT OF INTEREST STATEMENT

The authors declare no conflict of interest.

## ETHICS STATEMENT

All patients were informed about the study and consented to participate.

## Data Availability

The data set analysed in this study is available from the corresponding author on reasonable request.
